# Obesity is not associated with progression to end stage renal disease in patients with biopsy-proven glomerular diseases

**DOI:** 10.1186/s12882-019-1434-7

**Published:** 2019-07-02

**Authors:** Benjamin M. P. Elyan, Jennifer S. Lees, Keith A. Gillis, Bruce Mackinnon, Jonathan G. Fox, Colin C. Geddes, Emily P. McQuarrie

**Affiliations:** 1 Glasgow Renal and Transplant Unit, Queen Elizabeth University Hospital, Glasgow, UK; 2 Institute of Cardiovascular and Medical Sciences, University of Glasgow, Glasgow, UK

**Keywords:** Body mass index, Obesity, CKD, Risk factor, Glomerular disease

## Abstract

**Background:**

Body mass index (BMI) is associated with renal disease progression in unspecified CKD. The relationship between BMI and primary glomerular disease (GN) may be more complex. We aimed to evaluate the association between BMI and renal disease progression in patients with primary glomerular disease (GN).

**Methods:**

This was a single-centre retrospective cohort study performed in adult patients with biopsy-proven primary GN (excluding minimal change disease) from January 2000 to December 2015, with follow-up data until June 2017. BMI at time of biopsy was categorised as ≤25 kg/m^2^, > 25 to ≤30 kg/m^2^ and > 30 kg/m^2^. We used univariate and multivariate survival analyses to evaluate factors associated with progression to a composite endpoint of stage 5 CKD or renal replacement therapy (Major Adverse Renal Event - MARE) censoring for competing risk of death using Fine and Gray subdistribution hazards model.

**Results:**

We included 560 patients with biopsy-proven primary GN and available BMI data: 66.1% were male with median age 54.8 (IQR 41.1–66.2) years and BMI 28.2 (IQR 24.9–32.1) kg/m^2^. Those with BMI 25-30 kg/m^2^ (*n* = 210) and with BMI > 30 kg/m^2^ (*n* = 207) were older (*p* = 0.007) with higher systolic and diastolic blood pressures (*p* = 0.02 and 0.004 respectively) than those with BMI < 25 kg/m^2^ (*n* = 132). There was a greater proportion of focal segmental glomerulosclerosis in those with higher BMI (3.9% in BMI < 25 kg/m^2^, 7.9% in BMI 25–30 kg/m^2^ and 10.7% in BMI > 30 kg/m^2^ of biopsies (*p* = 0.01)), but similar proportions of other GN diagnoses across BMI groups. Baseline eGFR (*p* = 0.40) and uPCR (*p* = 0.17) were similar across BMI groups. There was no interaction between BMI and time to MARE (log-rank *p* = 0.98) or death (log-rank *p* = 0.42). Censoring for competing risk of death, factors associated with progression to MARE were: younger age, lower baseline eGFR and higher uPCR, but not BMI (SHR 0.99, 95%CI 0.97–1.01, *p* = 0.31) nor blood pressure or GN diagnosis.

**Conclusion:**

BMI was not associated with progression to MARE in this patient cohort with primary GN. Efforts should be directed to managing other known risk factors for CKD progression.

## Background

The proportion of overweight and obese patients has increased dramatically in recent years. The World Health Organisation quotes a three-fold increase in prevalence of obesity worldwide from 1975 to 2016 affecting up to 1.9 billion adults [1].

Body mass index (BMI) is widely and routinely used to assess relative adiposity, categorising patients as underweight, normal, overweight and obese. Obese and overweight individuals have been shown to have an increased propensity to develop a multitude of comorbidities involving the cardiovascular, respiratory, endocrine, musculoskeletal systems [2]. Obesity has also been shown to increase mortality rates in a variety of conditions [3]. There has been an increase in the proportion of overweight or obese adults in Scotland from 1995 to 2016 (52 to 65%) [4]. Obesity is a potentially modifiable risk factor for developing and treating these conditions.

The relationship between body weight and the kidney appears to be more complex. Obesity is a risk factor for incident chronic kidney disease (CKD) [5]. In two large cohort studies, elevated BMI was an independent risk factor for progression to both diabetic and non-diabetic End Stage Renal Disease (ESRD) amongst adolescents [6] and adults [7]. However, the association between BMI and progressive renal dysfunction follows a ‘U-shaped’ relationship with poor renal outcomes in underweight as well as overweight individuals [8]. Furthermore, being overweight or obese is associated with improved outcomes in ESRD, especially in the haemodialysis population. This is often referred to as the “obesity paradox” and has been replicated in many studies [9].

Obesity is associated with specific renal pathological changes including glomerulomegaly and thickening of the glomerular basement membrane, which can exist alongside other primary glomerulonephritis (GN) [10] or progress to overt focal segmental glomerulosclerosis (FSGS) [11]. There are some data describing outcomes in patients with primary GN according to BMI. This is best studied in IgA Nephropathy, where obese patients are less likely to go into spontaneous remission and more likely to have progressive renal disease [12–14]. Whether this is an independent effect or the result of confounding co-factors is unclear [15, 16]. Renal biopsy data from a Japanese registry study demonstrate that elevated BMI is associated with proteinuria in minimal change disease and membranous GN, but blood pressure and renal function were more important predictors of proteinuria in other glomerular diseases [17].

Managing risk factors for progression to renal endpoints is a vital aspect of managing patients with primary GN. Improved knowledge of the significance of these risk factors is central to this. We aimed to analyse the relationship between BMI and progression to renal endpoints in patients with biopsy-proven primary GN. We hypothesised that a higher BMI would be associated with progression of renal disease.

## Methods

In this cohort study, we included all adults (age >/= 16 years) who underwent native renal biopsy in a single centre between 01/01/2000 and 31/12/2015 with available BMI data. Follow-up data were available until 20/06/2017. Biochemical and anthropometric data were extracted from the electronic patient record. Demographic data included date of birth, gender and postcode. Deprivation was estimated using the Scottish Index of Multiple Deprivation (SIMD) in quintiles (SIMD 1 = greatest deprivation). The SIMD is produced using Scottish Government Statistics and gives a relative score of deprivation, based on multiple indicators of poverty and inequality for a given post code [18]. The height and weight at the time of biopsy were collected along with systolic and diastolic blood pressure. BMI was calculated from the standard equation: weight (kg)/(height (metres))^2^ and categorised into groups: BMI ≤25 kg/m^2^(normal weight), > 25 to ≤30 kg/m^2^ (overweight) and > 30 kg/m^2^ (obese).

The biopsy date, indication and diagnosis were extracted. The following laboratory parameters were collected at the time of biopsy: serum creatinine, serum albumin, estimated glomerular filtration rate (eGFR) by CKD-EPI and urinary protein to creatinine ratio (uPCR).

We defined the renal endpoints of interest as time from biopsy to chronic kidney disease Stage 5 (CKD 5- eGFR < 15 ml/min/1.73 m^2^) and/or renal replacement therapy (RRT). The dates of reaching CKD 5, RRT or death were recorded.

**Fig. 1:**
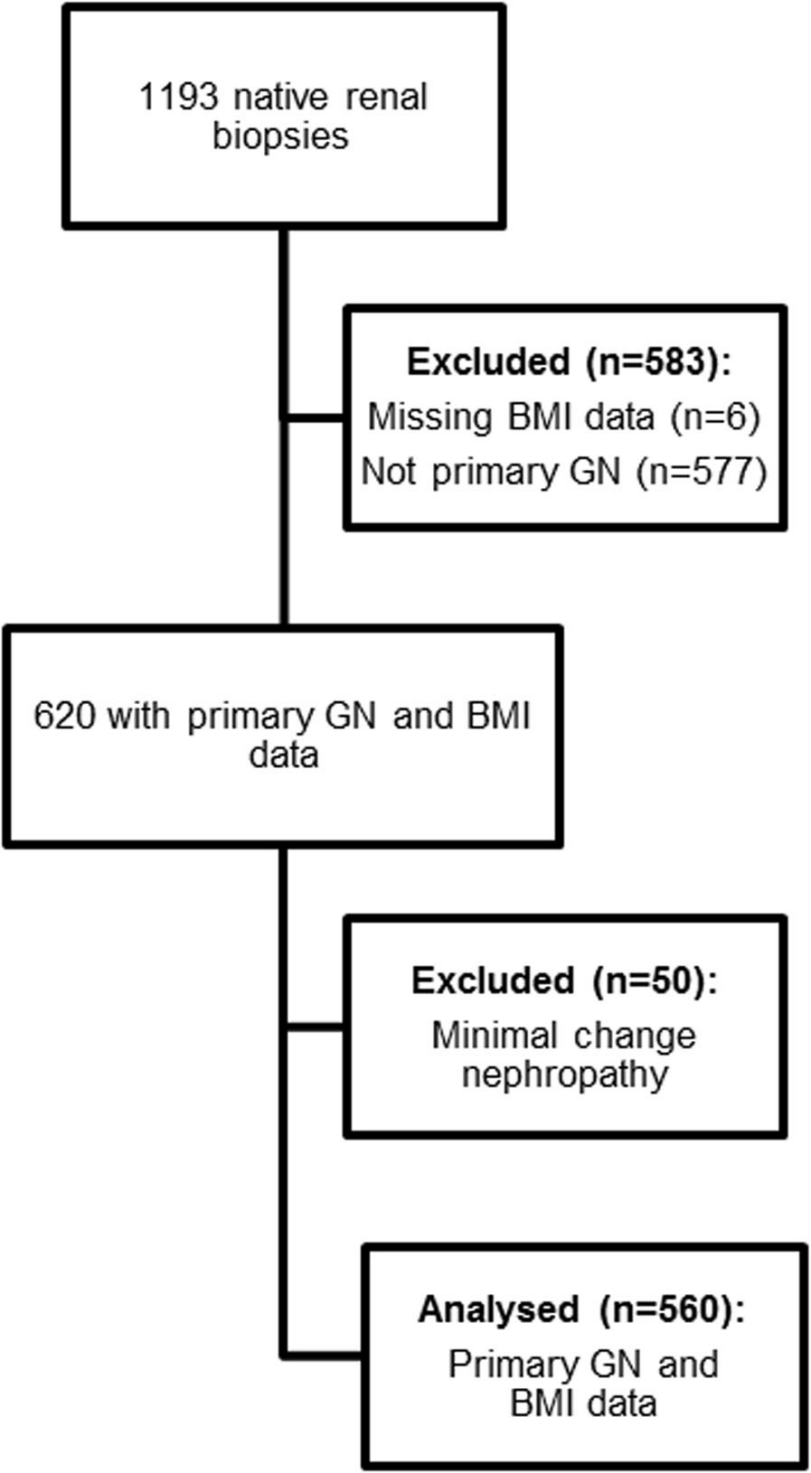
Consort diagram of included/excluded patients

Patients were excluded if they had no available weight or height records at the time of biopsy. Patients with a primary glomerular disease were categorised as membranous glomerulonephropathy (MGN), IgA nephropathy (IgAN), focal segmental glomerulosclerosis (FSGS), minimal change nephropathy (MCN) and other primary glomerular diseases (Other) for comparison. Patients with MCN were excluded, as this is not a progressive condition. (Fig. 1). Patients with primary diabetic nephropathy were excluded, but those considered to have a primary GN and background features of diabetic nephropathy were included.

Missing data (considered to be missing at random) were multiply imputed by chained equations [19]. The proportion of missing data for uPCR was 29.2%, and for all other variables was less than 5%. Five separate datasets were multiply imputed, and averaged across each other, before being entered into a survival analysis.

We performed unadjusted survival analysis according to BMI category (normal vs. overweight vs. obese) and compared groups using the Log-rank test. Subsequent multivariable survival analysis was performed to evaluate the factors independently associated with progression to a composite renal endpoint comprising CKD 5 or renal replacement therapy (MARE = major adverse renal event), adjusting for the competing risk of death using the Fine and Gray subdistribution hazards [20] and Cox cause-specific hazards models [21]. BMI was entered into the analyses both as a continuous variable and then by BMI group. Pearson’s coefficient was used to assess correlation between variables.

As a surrogate marker of fluid status (and therefore total body weight) at time of biopsy, we assessed change in weight after biopsy in those patients who were biopsied for nephrotic syndrome versus other indication. For each patient, up to 50 post-biopsy weight recordings were extracted. The weight closest to 6 months post-biopsy was used to calculate change in weight. We assumed that weight loss in the nephrotic group reflects correction of fluid overload. Summary data are presented as mean ± standard deviation (SD) or median and interquartile range (IQR) where data are not normally distributed. Between group comparisons of continuous data were evaluated using analysis of variance (ANOVA), whilst categorical data were evaluated using Chi square testing.

Analyses were conducted using *crr, mice, riskRegression, cmprsk* and *cr17* packages for R statistical software. Analyses were conducted in R Studio (version 1.0.136) available at http://www.R-project.org and distributed under the GNU (http://www.gnu.org) General Public License.

## Results

There were 1193 patients who underwent native renal biopsy between 01/01/2000 and 31/12/2015: complete BMI data were available in 1187 cases. Five hundred and sixty patients with biopsy-proven primary GN - excluding MCN - were included (Fig. 1). Baseline characteristics are described in Table 1.

**Table 1:** Summary table of baseline characteristics across BMI groups

Baseline characteristics	BMI < 25 (*n* = 143)	BMI 25–30 (*n* = 210)	BMI > 30 (*n* = 207)	All (*n* = 560)
Age (years): median (IQR)	48.1 (33.0–66.6)	55.7 (42.0–66.5)	55.0 (45.5–66.1)	54.8 (41.1–66.2)
Male (%)	61.6	72.0	63.3	66.1
BMI (kg/m^2^): median (IQR)	22.5 (20.9–23.8)	27.2 (26.0–28.6)	33.7 (31.6–36.8)	28.2 (24.9–32.1)
Systolic blood pressure (mmHg)	141 (23)	144 (22)	147 (21)	144 (22)
Diastolic Blood Pressure (mmHg)	75 (9)	80 (11)	82 (11)	80 (11)
eGFR (ml/min/1.73m^2^)	56 (38)	47 (31)	52 (34)	51 (34)
uPCR (mg/mmol): median (IQR)	318 (142–592)	317 (155–717)	400 (155–776)	337 (151–714)
Died during follow up: n(%)	40 (28.0)	50 (23.8)	43 (20.8)	133 (23.8)
RRT during follow up: n(%)	47 (32.9)	58 (27.6)	56 (27.1)	161 (28.9)
CKD 5 during follow up: n(%)	55 (38.5)	81 (38.6)	79 (38.2)	215 (38.4)
CKD 5 or RRT during follow up: n(%)	60 (42.0)	88 (41.9)	82 (39.6)	230 (41.1)
SIMD quintile: median (IQR)	2 (1–3)	2 (1–3)	2 (1–3)	2 (1–3)

*BMI* Body Mass Index (kg/m^2^), uPCR Urinary Protein Creatinine Ratio, eGFR estimated glomerular filtration rate, RRT Renal Replacement Therapy, CKD Chronic Kidney Disease, SIMD Scottish Index of Multiple Deprivation, All data are mean (standard deviation) unless otherwise indicated

There was a male preponderance (66.1%), with median age 54.8 (41.1–66.2) years and BMI at biopsy 28.2 (24.9–32.1) Kg/m^2^. Four patients were found to be underweight (BMI < 18 kg/m^2^) and were included in the category of patients with BMI < 25 kg/m^2^ (*N* = 143). Those with BMI 25–30 kg/m^2^ (*n* = 210) and > 30 kg/m^2^ (*n* = 207) were older than those with BMI < 25 kg/m^2^ (*p* = 0.007). In total 215 patients progressed to CKD 5 during follow up, 161 required RRT, and 230 patients reached CKD 5 or RRT (MARE). The total number of patients who died during follow up was 133.

**Fig. 2:**
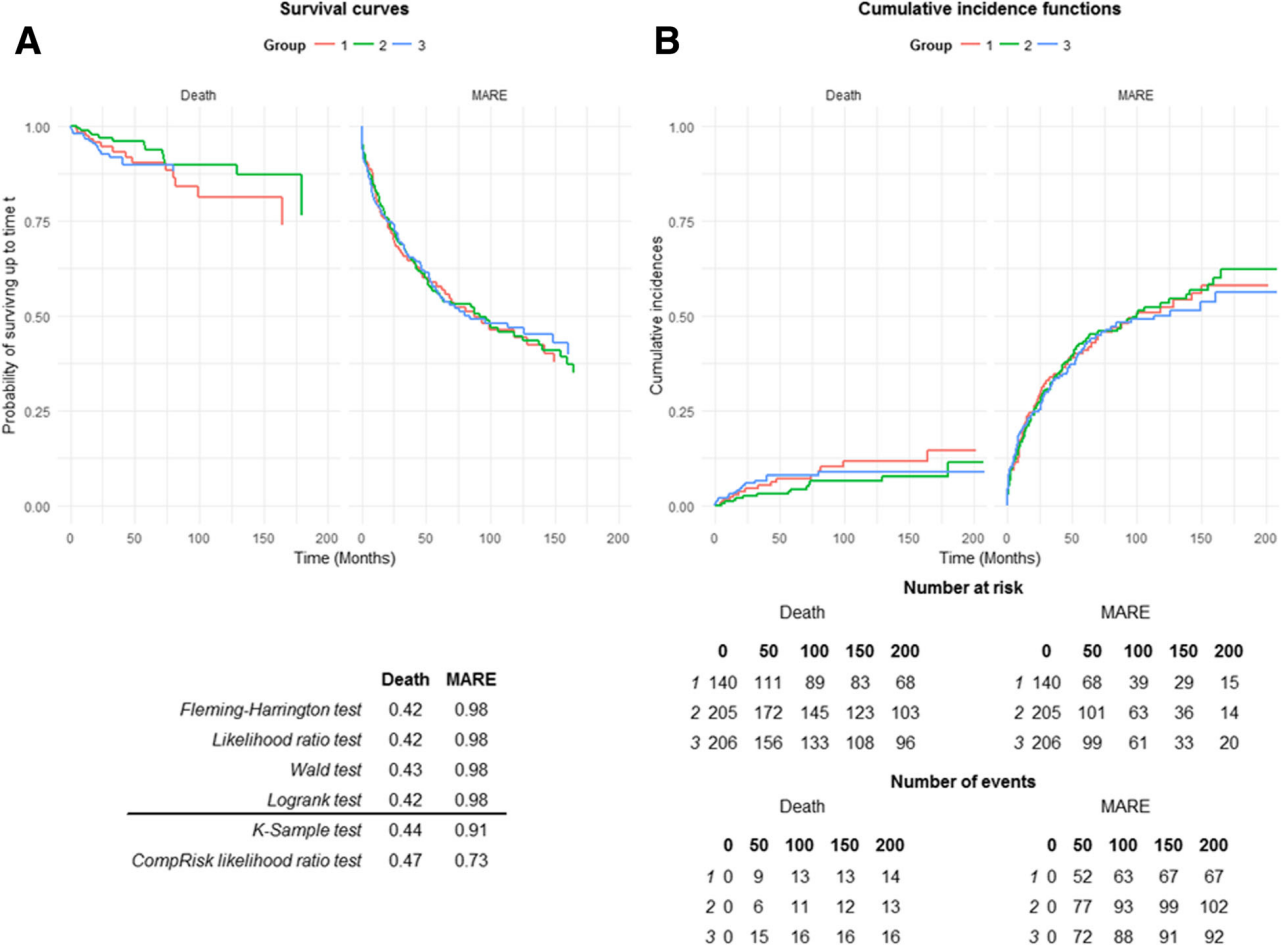
Survival curves (**a**) and cumulative incidence functions (**b**) of time to death and major adverse renal event (MARE) according to BMI group (1 – BMI < 25 kg/m^2^, 2 – BMI 25–30 kg/m^2^, 3 – BMI > 30 kg/m^2^)

The proportion of FSGS diagnosed increased across BMI categories: 3.9% in BMI < 25 kg/m^2^, 7.9% in BMI 25–30 kg/m^2^ and 10.7% in BMI > 30 kg/m^2^ of biopsies (*p* = 0.01), but similar proportions of other GN diagnoses across the groups (Table 2). BMI was positively correlated with systolic blood pressure (SBP) (*r* = 0.13, *p* < 0.01) and SIMD (r = 0.13, p = 0.01), but not eGFR (*r* = − 0.02, *p* = 0.58) or uPCR (r = 0.10, *p* = 0.06). There was no interaction between BMI and time to MARE (log-rank *p* = 0.98) or death (log-rank *p* = 0.42) (Fig. 2).

**Table 2:** Primary glomerular disease across BMI groups

Primary Glomerular Disease	Group 1: BMI < 25 (n = 143)	Group 2: BMI 25–30 (n = 210)	Group 3: BMI > 30 (n = 207)	Chi-square p=	Chi-square across groups
Membranous glomerulonephritis (%)	5.4	8.6	8.0	0.94	0.08
IgA nephropathy/HSP nephritis (%)	11.1	16.1	13.4	0.27	
Focal segmental glomerulosclerosis (%)	3.9	7.9	10.7	0.01	
Other primary glomerular disease (%)	5.2	5.0	4.8	0.23	

*BMI* Body Mass Index (kg/m^2^), *IgAN* IgA Nephropathy, *HSP* Henoch**-**Schonlein Purpura

Factors associated with progression to MARE, censoring for competing risk of death, included younger age (SHR 0.99 per 1-year increase in age, 95% CI 0.97–0.99, p = 0.01), lower eGFR (SHR 0.81 per 10 ml/min/1.73m^2^ increase in eGFR, 95% CI 0.76–0.86, *p* < 0.001) and higher uPCR (SHR 1.07 per 100 mg/mmol increase in uPCR, 95% CI 1.02–1.11, *p* = 0.002). There was no association of BMI, systolic blood pressure or primary glomerular diagnosis with progression to MARE. Subdistribution and cause-specific hazards of progression to MARE for each factor are illustrated in Table 3.

**Table 3:** Subdistribution and cause-specific hazard ratios for progression to major adverse renal event

Variable	Subdistribution hazard ratio	P	Cause-specific hazard ratio	P
Age per year increase	0.99 (0.98–1.00)	0.009	0.99 (0.98–1.00)	0.14
SBP per 10 mmHg increase	1.05 (0.98–1.11)	0.16	1.03 (0.97–1.09)	0.34
BMI (continuous– kg/m^2^)	0.99 (0.97–1.01)	0.31	1.01 (0.99–1.03)	0.37
BMI < 25 kg/m^2^	REF	–	REF	–
BMI 25–30 kg/m^2^	0.90 (0.65–1.24)	0.51	0.84 (0.61–1.15)	0.27
BMI > 30 kg/m^2^	0.83 (0.59–1.17)	0.28	0.88 (0.63–1.22)	0.44
eGFR per 10 ml/min/1.73m^2^ increase	0.81 (0.75–0.86)	< 0.001	0.80 (0.75–0.85)	< 0.001
uPCR per 100 mg/mmol increase	1.07 (1.02–1.11)	0.002	1.06 (1.04–1.09)	< 0.001
IgAN vs MGN	1.16 (0.75–1.81)	0.50	1.30 (0.85–1.97)	0.23
FSGS vs MGN	1.19 (0.77–1.84)	0.44	1.20 (0.78–1.83)	0.40
Other vs MGN	1.35 (0.83–2.18)	0.23	1.55 (0.98–2.46)	0.06
SIMD 2 vs 1	0.86 (0.62–1.18)	0.35	0.76 (0.55–1.06)	0.11
SIMD 3 vs 1	0.81 (0.54–1.21)	0.29	0.82 (0.56–1.21)	0.32
SIMD 4 vs 1	0.96 (0.63–1.46)	0.84	0.95 (0.64–1.42)	0.80
SIMD 5 vs 1	1.06 (0.67–1.66)	0.81	1.12 (0.73–1.72)	0.60

*BMI* Body Mass Index, *eGFR* estimated glomerular filtration rate, *MGN* Membranous Glomerulonephritis, *IgAN* IgA Nephropathy, *FSGS* Focal Segmental Glomerulosclerosis, *Other* Other Primary Glomerular Diagnosis, *uPCR* Protein Creatinine Ratio, *SIMD* Scottish Index of Multiple Deprivation

Change in weight in the 6-month period after biopsy was greater in nephrotic (− 2.1 kg; IQR -7.35 - 1.1) versus non-nephrotic patients (+ 0.2 kg; IQR -3.3 – 3.7). Change in weight according to BMI category (Table 4) or GN category (Table 5) shows greater weight loss in those with BMI > 30 kg/m^2^. If we assume that weight loss in nephrotic patients represents correction of fluid overload after biopsy, BMI category at biopsy was overestimated in 26 patients: 9 originally included in BMI category 25–30 kg/m^2^ and 17 originally with calculated BMI > 30 kg/m^2^.

**Table 4:** Weight and weight change at 6 months after biopsy-proven diagnosis across BMI groups

Values	Group 1 BMI < 25	Group 2 BMI 25–30	Group 3 BMI > 30	All
Number	139	198	195	532
Weight (kg)	64.3	80.2	100.4	83.4
BMI (kg/m^2^)	22.1	27.4	34.9	28.8
Weight change (kg)	0.7	−0.6	−2.0	−0.8

*BMI* Body Mass Index (kg/m^2^)

**Table 5:** Table demonstrating average weights and weight changes across primary glomerular disease categories

Values	MGN	IgAN	FSGS	Other	All
Number	118	218	117	79	532
Weight (kg)	83.5	83.2	87.4	78.2	83.4
BMI (kg/m^2^)	28.9	28.3	30.2	27.7	28.8
Weight change (kg)	−1.4	0.2	−1.6	−1.5	−0.8

*MGN* Membranous Glomerulonephritis, *IgAN* IgA Nephropathy, *FSGS* Focal Segmental Glomerulosclerosis, *Other* Other Primary Glomerular Diagnosis, *BMI* Body Mass Index, *kg* Kilogram

## Discussion

Obesity is a growing global health issue that has been shown to impact negatively on a multitude of factors regarding the health and wellbeing of patients. In this single centre study of a large cohort of patients with biopsy proven glomerular disease, we analysed the relationship of clinical and biological parameters with progression to renal endpoints, with focus on BMI as a risk factor. Two thirds of patients were overweight or obese, which is similar to the general population from which the cohort is derived [4]. BMI is correlated with known risk factors for renal progression (systolic blood pressure) but is not independently associated with progression to a combined renal endpoint of CKD 5 or RRT in this patient cohort. We have accounted for the main competing risk (death), recognised as an important consideration in survival analysis [22].

There are many proposed mechanisms by which obesity may have a direct effect on kidney function. Some studies have found that increasing fat consumption in mice results in increased proteinuria, an increase in glomerular tuft area, and mesangial expansion [23] and associated pathophysiological findings [24]. Increased renal blood flow in obesity has been demonstrated in both animal [25] and human studies [26]. Others hypothesise that increased adipose tissue increases circulating levels of adiponectin and leptin [27], inflammatory proteins, oxidative stress [28], insulin and insulin resistance and abnormalities in lipid metabolism [29]. These mechanisms potentially lead to glomerular hyperfiltration, glomerular hypertension and glomerulomegaly, thereby causing the proposed ‘obesity related glomerulopathy’. We observed no significant independent impact of BMI on renal progression in this cohort of patients with primary GN. It may be that in clinical practice, obesity exerts a deleterious effect on renal function via its association with higher blood pressure, rather than its other metabolic effects; as such strict control of hypertension may abrogate the effect of BMI on renal dysfunction. Similarly, various other lifestyle factors important in renal disease progression, such as physical activity [30] [31] and smoking status [32], may be associated with obesity; we cannot comment on such lifestyle factors in this analysis. Obesity and the metabolic syndrome, including insulin resistance and hyperglycaemia, is likely to play a more complex role in the pathophysiology of diabetic nephropathy than in other glomerulopathies. Diabetic nephropathy is the single commonest cause of renal failure and accounts for nearly 28% of ESRD in the Scotland [33]. Exclusion of patients with primary diabetic and hypertensive nephropathy in our study may explain the difference between the observed impact of BMI in undifferentiated CKD and in primary GN.

It is interesting that in our cohort, FSGS is more common in patients with a BMI > 30, but that other diagnoses are also equally represented. This finding is supported by recent data from Columbia University: amongst 248 kidney biopsies performed in patients with BMI > 40 kg/m^2^, obesity-related glomerulopathy (glomerulomegaly +/− FSGS) alone was found in 29% of patients, and the other 71% had other kidney disease without evidence of obesity-related glomerulopathy [34]. This emphasises the importance of considering native renal biopsy in obese patients and not assuming an obesity-related phenomenon.

Our study has some limitations. First, BMI is a widely used but imperfect tool to assess body fat: it does not consider body composition (including fluid status), age or gender, and may underestimate the obese and underweight categories, particularly in men [35]. Other assessment tools of obesity have been proposed, such as percentage body fat, waist circumference or hip to waist ratio. It has been demonstrated that reported differences between BMI and these assessments are “too small to be of clinical relevance” and that BMI is favourable due to the ease of collection and extraction of the data required [36]. Second, BMI may have been overestimated in those patients with nephrotic syndrome or fluid overload. In those patients, if the observed weight loss in the first 6 months accurately represents fluid overload at time of biopsy, calculated BMI would have overestimated BMI category for 26 patients (9 with calculated BMI 25-30 kg/m^2^ and 17 with BMI > 30 kg/m^2^). However, this would also have overestimated the potential impact of BMI on renal disease and we think it unlikely this has unduly influenced our results. Third, we are unable to comment on the effect of being underweight (BMI < 18 kg/m^2^) on renal progression because of the small number of patients in this category. Fourth, due to relatively small sample size, we have combined data from patients with any primary glomerular disease, and thus cannot comment on whether there is any influence of BMI on progression to renal endpoints in individual disease processes, such as FSGS, which we have shown to be associated with obesity. Fifth, we are unable to comment on the degree of pathological injury (glomerulosclerosis, interstitial fibrosis and tubular atrophy) on renal biopsy samples, but instead have used surrogate markers of renal injury (eGFR and proteinuria). Sixth, due to the retrospective design of the study, there may be other confounders for which we have not adjusted. Last, these findings are limited to a predominantly Caucasian population.

## Conclusions

In this cohort of patients with biopsy-proven primary GN, there was no association between BMI and progression to major adverse renal event of CKD stage 5 or requirement for renal replacement therapy. FSGS was more common in overweight or obese patients, but other diagnoses are equally represented; this supports pursuit of pathological diagnosis of renal disease in patients who are obese. Without evidence to support weight loss in improving renal outcomes in these patients, we recommend that clinicians focus on improving management of known risk factors for renal progression, such as hypertension, proteinuria and smoking cessation.

## Data Availability

The datasets generated during the current study are not publicly but are available from the corresponding author on reasonable request.

## Abbreviations

BMI: Body mass index
CKD: Chronic kidney disease
eGFR: estimated glomerular filtration rate
ESRD: End-stage renal disease
FSGS: Focal segmental glomerulosclerosis
GN: Glomerulonephritis
MARE: Major adverse renal event
MCN: Minimal change nephropathy
MGN: Membranous glomerulonephropathy
RRT: Renal replacement therapy
SIMD: Scottish Index of Multiple Deprivation
uPCR: urinary protein to creatinine ratio
